# Supplementary material for: Essential Oil from Glossogyne tenuifolia Inhibits Lipopolysaccharide-Induced Inflammation-Associated Genes in Macro-Phage Cells via Suppression of NF-κB Signaling Pathway
Source: Plants (Basel). 2023 Mar 9;12(6):1241. doi: 10.3390/plants12061241 (PMC10054403; doi:10.3390/plants12061241)
Supplement: Supplementary file 1 [file plants-12-01241-s001.zip › plants-2250458-supplementary.pdf]

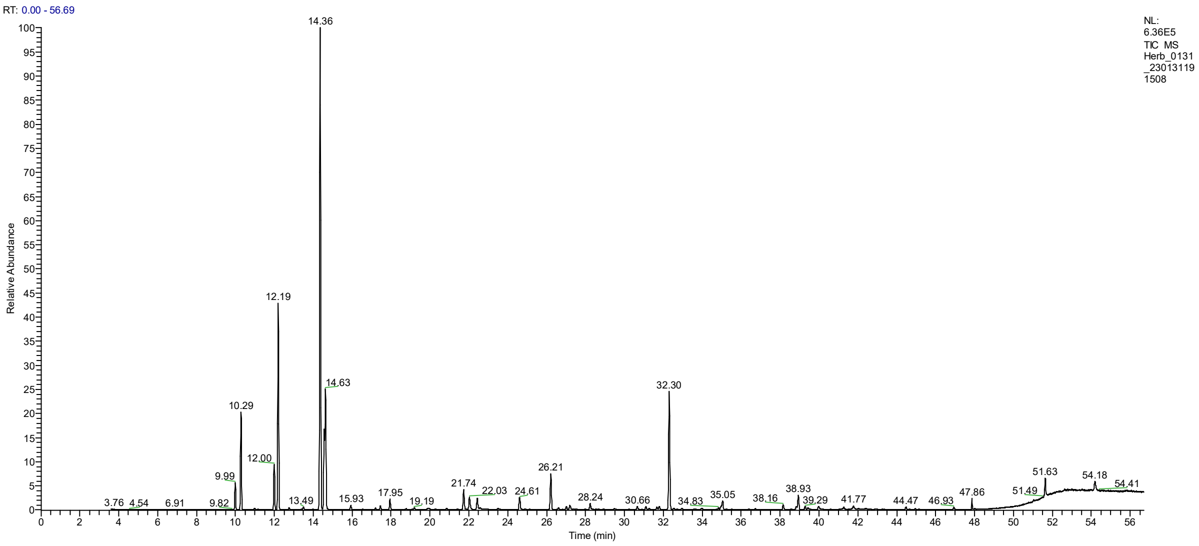

Figure S1. GC-MS spectrum of *Glossogyne tenuifolia* essential oil.

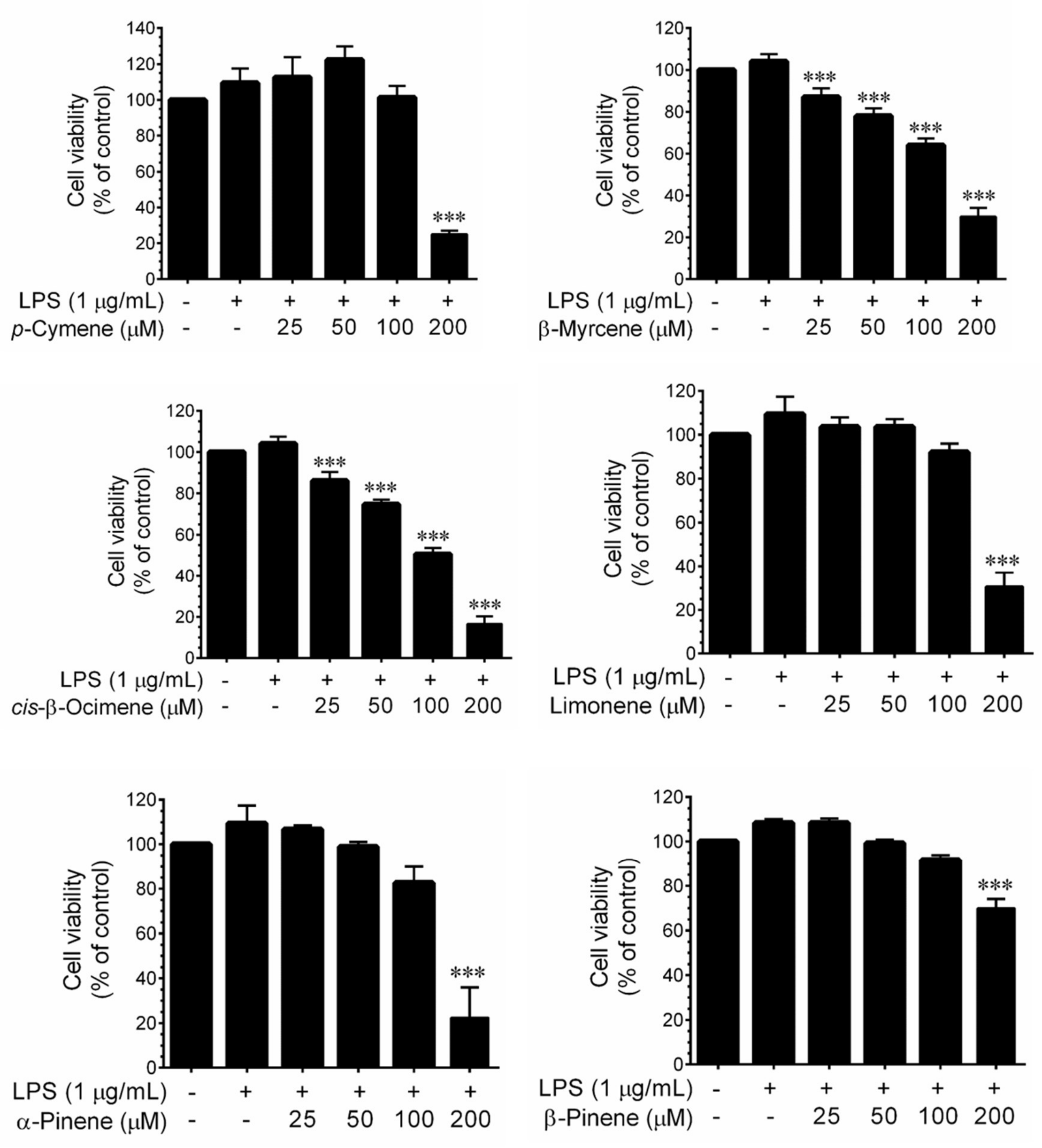

Figure S2. Effect of *p*-cymene, β-myrcene, cis-β-ocimene, α-pinene, β-pinene, and limonene on cell viability. RAW 264.7 cells were incubated with increasing concentrations of test compounds (25–200 μM) in the presence of 1 μg/mL LPS for 24 h. The cell viability was determined by the MTT colorimetric assay. Data are reported as mean ± SD of three independent experiments. $^{***}P < 0.001$ indicates a significant difference between the control and test samples treated groups.
